# Scale for the Assessment and Rating of Ataxia (SARA): Development of a Training Tool and Certification Program

**DOI:** 10.1007/s12311-023-01543-3

**Published:** 2023-03-15

**Authors:** Marcus Grobe-Einsler, Arian Taheri Amin, Jennifer Faber, Hartmut Völkel, Matthis Synofzik, Thomas Klockgether

**Affiliations:** 1 German Center for Neurodegenerative Diseases (DZNE), Venusberg-Campus 1, D-53127 Bonn, Germany; 2 Department of Neurology, University Hospital Bonn, Bonn, Germany; 3 Department of Neurology, University Hospital Tübingen, Tübingen, Germany; 4 German Center for Neurodegenerative Diseases (DZNE), Tübingen, Germany

**Keywords:** Ataxia, SARA, Certification, Training tool

## Abstract

Monitoring of disease severity is of great importance for treatment and management of clinical trials. The Scale for Assessment and Rating of Ataxia (SARA) is a frequently used, short and easily applicable clinical scale used to assess the severity of ataxia. The objective of our study was to develop a training and certification tool for the SARA. SARA scores were recorded according to a standardized protocol and rated by three clinical experts in consensus. Four hundred thirty-eight videos of 67 patients were included in the SARA training tool. The tutorial section demonstrates a complete SARA examination on a healthy control. In the training section, users can compare their ratings to consensus ratings and access a video library covering the complete SARA range. The tool also includes a section that allows optional certification. The SARA training tool provides comprehensive and standardized training material and certification to reduce variability in applying the SARA. Standardization aims to improve the quality of patient care and research in ataxia.

**Supplementary Information:**

The online version contains supplementary material available at 10.1007/s12311-023-01543-3.

## Introduction

Ataxia means ‘absence of order’ and denotes the clinical syndrome of imbalance, disturbed coordination, and slurred speech. It arises due to any focal or diffuse damage to the cerebellum and its afferent and efferent connections and can be encountered in numerous neurological conditions including stroke, multiple sclerosis, encephalitis, and brain tumors [1]. Ataxia also refers to a specific group of neurodegenerative diseases of the nervous system in which progressive ataxia is the prominent clinical manifestation. The ataxias comprise diseases of both genetic and non-genetic origin [2, 3].

Reliable assessment of the severity of ataxia and its changes over time by use of an appropriate clinical scale is of outstanding importance both for patient management and for research purposes. In patients with acquired ataxias who undergo specific treatments, e.g. immune suppression in paraneoplastic ataxia, monitoring of ataxia severity is mandatory. Measuring ataxia severity is likewise important in assessment of the success of rehabilitation in patients with cerebellar strokes. In clinical research, precise assessment of ataxia severity is indispensable both, in natural history studies and even more in interventional trials.

The Scale for the Assessment and Rating of Ataxia (SARA) is a short and easy to use clinical score to assess the core symptoms of ataxia. It consists of 8 items (gait, stance, sitting, speech disturbance, finger-chase, nose-finger test, fast alternating hand movements and heel-shin slide) that sum up to a maximum of 40 points with higher scores indicating more severe ataxia [4]. The SARA has been carefully validated in different kinds of ataxia and is widely used in movement disorder clinics, rehabilitation centers, and for clinical studies [5–7].

To obtain meaningful and reliable results on ataxia severity, correct application of the SARA is essential. Although the SARA instructions are very precise, they sometimes leave room for discussion on how to correctly perform and rate single items. This increases inter-rater variability especially in multicentric studies. This effect is aggravated by a lack of standardized teaching material or certification for application of the SARA. Standardized video-based teaching material and certification programs already exist for a number of different clinical scores, including the unified Parkinson disease rating scale (UPDRS) [8] and the unified Huntington disease rating scale (UHDRS) [9], and diagnosis criteria, for example for progressive supranuclear palsy [10]. Application of teaching videos and certification programs serves to reduce inter-rater variability in clinical trials, but also provides a guideline on correct performance in routine application [11, 12]. In this study, we develop a video-based training and certification program for the SARA.

## Materials and Methods

### Recordings and Ratings

All videos were recorded according to a standardized protocol during consecutive routine study visits in ongoing observational trials in the German Center for Neurodegenerative Diseases (DZNE) in Bonn. The study was approved by the local ethics committee and all patients gave written consent to participation. Some recordings, including the finger-nose test (item 6) do not allow sufficient blurring of the face, so patients were particularly informed and consented that the recordings include potentially identifying features. Prescreening included quality control of videos and correct performance of the recorded items. In joint sessions, three ataxia experts (TK, MS, MGE) rated all recordings that passed quality control to obtain a consensus rating. Videos with ambiguous ratings were excluded from the training tool.

### Development of the Training Tool

The tool is divided into three Sections. (1) tutorial section, (2) training section and (3) certification. Based on clinical experience and examples from the rating process, the consensus raters identified possible pitfalls in the application of the SARA. All raters agreed on a standardized interpretation of the respective SARA instructions. Hints on how to avoid these pitfalls were included in the *tutorial section* of the training tool.

## Results

### Tutorial Section

The tutorial section of the SARA training tool consists of a recorded complete SARA examination of a healthy control. Recordings from real patients are not included in this section, because (1) severe ataxia usually hinders patients from performing a complete SARA (for example patients may only be able to perform one of the three stance tasks) and (2) in respect to data protection regulations, since we aim to make the tutorial video openly available.

### Training Section

This section includes all patient recordings selected by the consensus raters. A total number of 67 patients were rated. Individual SARA items contributed 536 videos; however, 98 videos were excluded, because they did not pass the quality control or had ambiguous consense ratings, resulting in 438 videos for the certification. Users can watch and rate all patient videos sorted by items and compare their own result with the consensus ratings. Additionally, users can access a SARA library with exemplary videos for all available SARA ratings. Use of this section is not restricted and users may choose to watch as many videos as needed.

### Certification Section

Users choosing to gain SARA certification will be asked to rate two SARA scores, excluding item 4, speech. This item has been excluded from the certification, because most of the recorded speech samples are in German. None of the recordings in this section is included in the tutorial and training section. After completion of the ratings, results will be compared to the consensus ratings. ≥ 80% correct ratings are prerequisite to obtain certification in this section.

### Availability and User Response

Certification is rarely required outside clinical trials, but standardized performance of the SARA is highly important to rate progression over time in clinical routine, especially if more than one investigator is involved, we aim to make the SARA tutorial section openly available for neurologists and health professionals working with ataxic patients (supplementary video). Availability of the complete certification program will remain limited to health care professionals working in the field of ataxia and participants of clinical and/or pharmaceutical trials with due regard to data protection regulations. The complete SARA training tool is currently embedded in DZNE’s e-learning platform DECLARE (access can be requested here: http://www.ern-rnd.eu/sara-training-tool-by-dzne/). The SARA training tool was first announced and made public to participants of the SCA Global & ARCA Global Joint Online Conference (19–21 Oct 2020). Since then, the training tool was used in 4 interventional trials, and 127 of 265 users decided to gain certification. In addition to this, we hereby make the tutorial section publicly available for health care professionals working with ataxic patients who want to learn how to apply the SARA or standardize their performance but may not require certification (supplementary video).

## Discussion

Despite the frequent use of the SARA in patient management and clinical studies, there is no standardized teaching material on how to apply the score, apart from a number of youtube videos. With the SARA training tool, we intend to close this gap. Upcoming interventional trials emphasize the need for such material.

The SARA instructions were written in English language and there are no official translations available. The SARA Training tool is therefore also provided in English, but translation of at least the tutorial section is easy to accomplish in the future. Since all recordings have been performed in Bonn, Germany, one limitation of the training program is the lack of availability of English speech samples. We therefore eliminated item 4 from the certification process, but included the German speech samples in the training section. Although video material is constantly used for teaching purposes, systematic effects of video ratings compared to on-site ratings have never been systematically investigated. This lack of correlating data represents another limitation that is shared among all video-based training tools. Based on the extensive video material obtained during the ongoing recordings, future and ongoing studies will investigate the inter-rater variability in video-based rating processes, as frequently used in observational and interventional trials today, and effects of rater training on rating performance in non-experienced SARA raters.

### Supplementary Information

Below is the link to the electronic supplementary material.

Supplementary file1 (MP4 732367 KB)

## Data Availability

Access to patient videos is restricted due to data protection issues. All remaining data that support the findings of this study are available from the corresponding author upon reasonable request.
